# Effects of Passion Flower Extract, as an Add-On Treatment to Sertraline, on Reaction Time in Patients ‎with Generalized Anxiety Disorder: A Double-Blind Placebo-Controlled Study

**Published:** 2016-07

**Authors:** Mandana Nojoumi, Padideh Ghaeli, Samrand Salimi, Ali Sharifi, Firoozeh Raisi

**Affiliations:** 1 **‎**Department of Clinical Pharmacy, Faculty of Pharmacy, Islamic Azad University of Pharmaceutical ‎Sciences, Tehran, Iran.; 2 Department of Clinical Pharmacy, Faculty of Pharmacy, Tehran University of Medical Sciences, ‎Tehran, Iran.; 3 Baharloo Hospital, Tehran University of Medical Sciences, Tehran, Iran.; 4 Research Centre for Intelligent Signal Processing, Tehran, Iran.; 5 Psychiatric and Psychology Research Centre, Roozbeh Psychiatric Hospital, Tehran University of ‎Medical Sciences, Tehran, Iran.

**Keywords:** *Generalized Anxiety Disorder*, *Mental Processing*, *Passion Flower*, *Reaction Time*, *Sertraline*‎

## Abstract

**Objective: **Because of functional impairment caused by generalized anxiety disorder and due to cognitive side ‎effects of many anti-anxiety agents, in this study we aimed to evaluate the influence of Passion ‎flower standardized extract on reaction time in patients with generalized anxiety disorder.‎

**Method: **Thirty patients aged 18 to 50 years of age, who were diagnosed with generalized anxiety disorder and ‎fulfilled the study criteria, entered this double-blind placebo-controlled study. Reaction time was ‎measured at baseline and after one month of treatment using computerized software. Correct ‎responses, omission and substitution errors and the mean time of correct responses (reaction time) in ‎both visual and auditory tests were collected. The analysis was performed between the two groups ‎and within each group utilizing SPSS PASW- statics, Version 18. P-value less than 0.05 was ‎considered statistically significant.‎

**Results: **All the participants were initiated on Sertraline 50 mg/day, and the dosage was increased to 100 ‎mg / day after two weeks. Fourteen patients received Pasipy (Passion Flower) 15 drops three times ‎daily and 16 received placebo concurrently. Inter-group comparison proved no significant difference ‎in any of the test items between assortments while a significant decline was observed in auditory ‎omission errors in passion flower group after on month of treatment using intra-group analysis.‎‎

**Conclusion: **This study noted that passion flower might be suitable as an add-on in the treatment of generalized ‎anxiety disorder with low side effects. Further studies with longer duration are recommended to ‎confirm the results of this study.‎

The Generalized Anxiety Disorder (GAD) is defined as the basic anxiety disorder, which may reflect ‎the fundamental process of all emotional disorders and significant degree of functional ‎impairment (1). GAD is hyper-reactivity and a fear of negative emotional shifts and ‎unmanageable worry about preventing these perceptive contrasts (2). The symptoms are difficult ‎to control and last for more than six months. GAD is associated with three or more of diagnostic ‎items from DSM-IV (Diagnostic and Statistical manual of Mental illnesses-4th edition) including: ‎Feeling keyed up or on edge, easily getting fatigue, mind going blank, agitation, somatic tension ‎and sleep disturbances. Treatment choices include psychological therapies such as cognitive ‎behavioral therapy (CBT) as the main non-pharmacological therapy (3), acceptance and ‎commitment therapy (4), intolerance of uncertainty therapy and motivational interviewing (5) as ‎well as pharmacotherapy including Selective Serotonin Reuptake Inhibitors (SSRIs) (6), Benzodiazepines (7), Pregabalin (8) and ‎Gabapentin (9), Tricyclic Antidepressants (TCAs), Buspirone and Hydroxyzine (6). Reaction Time (RT) is defined as the time ‎elapsed between offering stimuli and the indication of comprehension by the subject (10). RT is ‎claimed to be the main dependent variable for analyzing perceptive models (11).

Response procedure is directly based on circumstances (12). Many factors may be responsible for ‎reaction time fluctuations, specially a great number of drugs and substances e.g., Caffeine (13), ‎alcohol (14), psychostimulants (15), sedative-hypnotic and anti-epileptic drugs (16, 17) and many ‎of cognitive side effects, which are raised by psychiatric pharmacotherapies (18, 19).

‎Passion flower symbolizes the passion of Jesus in Christian theology because of its unique ‎structure (20).

‎Traditionally its extract has been used as an herbal remedy for nervous anxiety (21) and ‎insomnia, tenderness, restlessness, irritability (22) and hysteria (23). Passion flower has been ‎reported to affect GAD (24). Most of these effects are believed to be related to benzoflavone, ‎which is the active constituent of the plant extract (25). We aimed to investigate the effects of ‎passion flower extract on perceptual processing toward threats via reaction time test since its ‎advantage on mental function did not receive specific reflections in previous studies.‎‎

## Materials and Method

***Research Participants***

Thirty outpatients entered this randomized double-blind placebo-controlled study (Ethical ‎approval number 7408 - by Ethics Committee at Islamic Azad University of Pharmaceutical ‎Sciences). The participants were included in the study from Roozbeh and Baharloo hospitals and ‎private psychiatric offices during 2010- 2012. Patients were diagnosed with Generalized Anxiety ‎Disorder (GAD) based on DSM-IV criteria and clinical interviews. Their family history was ‎considered as well. They were tested using Hamilton Anxiety Rating Scale Form A (HARS). ‎Hamilton Rating Scale for Depression (HRSD) was utilized to determine the patients’ comorbid ‎depression. The Hamilton Scales were standardized for Iranian patients.

Patients between 18 to 24 years of age were included. In addition, sertraline consumption was ‎considered the best treatment for their current disease per decision of the psychiatrist. All patients ‎were initiated on Sertraline. The exclusion criteria were as follows: Having difficulty including ‎allergic reactions to sertraline or active ingredients of passion flower, renal or hepatic impairment, ‎age under 18, pregnancy and lactation, consuming Warfarin, Hexobarbital, Pantobarbital, ‎Levothyroxine or other thyroid medications, using alcohol or hallucinogens and history of ‎tachycardia. The patients with a history of kidney or liver dysfunction were excluded. An ‎informed consent was obtained from the patients prior to the initiation of the examination. ‎

***Medication***

The first-line treatment for GAD patients was 50 mg Sertraline tablet for both groups. Pasipy® Drop - Iran Darouk Co. was the standardized hydroalcoholic extract of passion flower as ‎an add-on therapy. 

Placebo consisted of 20% aqueous solution of absolute edible alcohol and natural coloring ‎agents. The placebo mixture was filled in amber glass bottles with dropper identical to the drug ‎container.‎

***Assortment***

The participants were randomly assigned into two groups to receive either Sertraline + Pasipy (S-‎drug group), or Sertraline + placebo (S-placebo group) for one month. All patients were initiated on ‎Sertraline 50 mg/day; the dosage was increased to 100 mg/day after two weeks. Pasipy and its ‎placebo were given at 15 drops three times daily.‎

***Data Collection Tools***

The Reaction Time (RT) test was utilized as the standard computerized software. These process ‎measured psycho-neural responses toward visual and auditory stimuli. The input variables ‎were the number of correct responses, omission and substitution errors and the mean time of ‎correct responses (mean reaction time) (26). After receiving each of the visual or auditory stimuli, the participants were asked to hit the correct keys, which were designed on a computer ‎keyboard. The sign on each key was related to a specific visual or auditory threat in the tests. The ‎ stimuli were presented continuously on the screen during the test procedure. Correct responses ‎were made when the participants had chosen the key that was the same as the presented stimulus, ‎whereas choosing an incorrect answer was considered as a substitution error. When the patient ‎ignored a visual or auditory stimuli, the answer was recorded as an omission error. Reaction ‎time was the mean time of correct responses to stimuli in each of the visual or auditory tests. ‎Test items were measured at baseline and after one month of S-drug or S-placebo administration. ‎A questionnaire of adverse effects or possible drug interactions was filled at the end of the study.‎

***Statistical Analysis***

Demographic characteristics were compared between the two groups. The RT test outputs were analyzed once in comparison between S-drug and S-placebo groups ‎using independent sample t-test (inter-group comparison); then reaction time changes after one ‎month was determined in each group using a paired sample t-test (intra-group comparison). ‎Scores from the Hamilton anxiety scale form A (HAM-A) were compared between S-drug and S-‎placebo groups using an independent sample t-test. The aim was to reconfirm the positive effect ‎of passion flower on GAD and the possible improvement of the add-on therapy encountered with ‎the SSRI monotherapy. All the comparisons were performed utilizing SPSS software (PASW – ‎statistics 18). A p-value of less than 0.05 was considered as the minimal level of statistical ‎significance in all measures.‎

## Results

Seventy patients were selected for the study; of whom, 24 were excluded as they did not meet our ‎criteria, and 16 did not follow the medication protocol because of low compliance and drug ‎incompatibility. The patients who met the inclusion criteria were randomized by permeated block ‎randomization (Table 1).‎

**Table1:** Demographic characteristics of patients in both groups

Group	**Sertraline + drug**	**Sertraline + placebo**	**P-value**
Item	**N = 14**	**N = 16**	
Age(mean±SD ¶)	29.07 ± 8.60	32.19 ± 11.43	0.410
Gender (Percent)	F: 85.7% - M: 14.3%	F: 87.5% - M: 12.5%	0.891
Caffeine intake (mg/day)	173.54 ± 99.17	130.46 ± 67.98	0.203

*: Significant difference (P-value < 0.05)

¶ SD: Standard deviation

**Table2:** Comparison of Reaction time parameters between the Two study Groups after One Month

**Group**	**Sertraline + drug N = 14** **‎**	**Sertraline + placebo N = 16** **‎**	**P-value**
Visual test	**mean ± SD** ¶	**mean ± SD**	
Correct responses	8.43 ± 6.00	9.69 ± 0.09	0.663
Substitution errors	10.57 ± 4.85	8.19 ± 4.04	0.153
Omission errors	11.00 ± 5.94	12.13 ± 7.90	0.666
Mean response time (second)	0.65 ± 0.12	0.64 ± 0.12	0.720
Auditory test	**mean ± SD**	**mean ± SD**	
Correct responses	3.64 ± 1.69	4.25 ± 3.51	0.561
Substitution errors	10.93 ± 5.84	8.44 ± 4.94	0.216
Omission errors	15.43 ± 6.76	17.31 ± 7.43	0.476
Mean response time (second)	0.49 ± 0.12	0.55 ± 12	0.467

*: Significant difference (P-value < 0.05)

¶ SD: Standard deviation

**Table3:** Comparison of Reaction time parameters within each study group at baseline (1) and after One Month (2)

Group	**Sertraline + drug**	**P-value**	**Sertraline + placebo**	**P-value**
Visual test	**Mean ± SD** ¶		**Mean ± SD**	
Correct responses 1	9.21 ± 7.99	0.555	10.69 ± 8.24	0.323
Correct responses 2	8.43 ± 6.00		9.69 ± 9.09	
Substitution errors 1	9.14 ± 3.84	0.222	8.50 ± 4.82	0.808
Substitution errors 2	10.57 ± 4.85		8.19 ± 4.04	
Omission errors 1	11.64 ± 6.79	0.585	10.81 ± 7.31	0.340
Omission errors 2	11.00 ± 5.94		12.13 ± 7.90	
Mean response time 1 (second)	0.59 ± 0.21	0.288	0.61 ± 0.21	0.549
Mean response time 2 (second)	0.64 ± 0.11		0.64 ± 0.12	
Auditory test	**mean ± SD**		**mean ± SD**	
Correct responses 1	3.36 ± 1.45	0.537	4.19 ± 4.86	0.939
Correct responses 2	3.64 ± 1.69		4.25 ± 3.51	
Substitution errors 1	8.71 ± 4.39	0.054	8.69 ± 4.76	0.845
Substitution errors 2	10.93 ± 5.84		8.44 ± 4.94	
Omission errors 1	8.71 ± 4.39	0.045 *	17.13 ± 6.11	0.898
Omission errors 2	15.43 ± 6.76		17.31 ± 7.43	
Mean response time 1 (second)	0.44 ± 0.16	0.484	0.60 ± 0.16	0.312
Mean response time 2 (second)	0.49 ± 0.21		0.54 ± 0.18	

* : Significant difference (P-value < 0.05)

¶ SD: Standard deviation

1 : baseline

2 ‎‎ : One month after drug or placebo consumption

**Table4:** Comparison of Hamilton Anxiety Rating Scale Form (HARS) at Baseline between the Two Study Groups

Group	**Sertraline + drug N = 14**	**Sertraline + placebo N = 16**	**P-value**
Score	**Mean ± SD ** ¶	**Mean ± SD**	
Baseline	21.54 ± 8.15	24.07 ± 10.73	0.495
After 1 month	16.44 ± 7.15	23.08 ± 8.85	0.039 *

* : Significant difference (P-value < 0.05)

¶ SD: Standard deviation

**Table5:** List of Reported Adverse Effects by Patients in Both Study Groups

Group	**Sertraline + drug**	**N = 14**	**Sertraline + placebo**	**N = 16**	**P-value**
Item	**Frequency**	**Percent**	**Frequency**	**Percent**	
Allergy	**1**	2.9 %	**1**	2.9 %	0.925
Asthma	**1**	2.9 %	**1**	2.9 %	0.925
Sinus irritation	**1**	2.9 %	**1**	2.9 %	0.925
Dermatitis	**1**	2.9 %	**3**	**8.8%**	**0.634**
Subcutaneous phlebitis	**0**	**0%**	**0**	**0%**	NS
Tachycardia	**3**	**8.8%**	**3**	**8.8%**	0.861
Nausea	**7**	**20.6%**	**3**	**8.8%**	**0.319**
Vomiting	**2**	**5.9%**	**0**	**0%**	**0.165**
Dizziness	**5**	**14.7%**	**2**	**5.9%**	0.155
Somnolence	**8**	**23.5%**	**7**	**20.6%**	**0.481**
Excessive sedation	**2**	**5.9%**	**1**	2.9 %	**0.493**
Abnormal bleeding	**0**	**0%**	**1**	2.9 %	0.333
etc.	**3**	**8.8%**	**4**	**11.8%**	**0.825**

Fourteen patients (85.7% female and 14.3% male) were initiated on Sertraline (50 mg/day and ‎the dosage was increased to 100 mg/day after two weeks) + Pasipy (15 drops three times daily). ‎The mean age ± standard deviation (SD) of these patients was 29.07 ± 8.60. Sixteen patients ‎‎(87.5% female and 12.5% male) were initiated on Sertraline (50 mg/day and the dosage was ‎increased to 100 mg/day after two weeks) + placebo (15 drops three times daily). The mean age in ‎this group was 32.19 ± 11.43.‎

***Inter-group analysis***

After one month, independent sample t-test did not demonstrate any significant difference in any ‎of visual or auditory items. Baseline scores were proved not to be statistically different, but they ‎are not displayed in the tables. In the visual test for the drug group, the omission errors were less ‎than the placebo consumers, but this difference was not statistically significant (P = 0.666). ‎However, the mean reaction time was slightly longer in this group (P = 0.720).

 ‎In auditory analysis for the drug group, omission errors were less than the placebo group, but the ‎difference was not significant (P = 0.476). However, the mean reaction time toward sound threats ‎improved slightly after one month of taking Pasipy in the drug group compared to the placebo ‎group (P = 0.467) (Table 2).‎

***Intra-group Analysis***

In the drug group, a significant decline in auditory omission errors was observed after one month ‎of treatment (P = 0.045). The mean reaction time had a non-significant increase in both visual (P ‎‎= 0.288) and auditory tests (P = 0.484) in drug intra-group analysis. None of the changes in test ‎variables in placebo consumers reached the significant level. The mean reaction time was a bit ‎longer in the visual test (P = 0.549), but had a non-significant improvement toward auditory ‎ stimuli in this group (P = 0.312) (Table 3).

***Hamilton Test***

Hamilton Anxiety Rating Scale Form A (HARS) questionnaires were ranged between 18 to 24 (mild to moderate). A significant improvement to relieve anxiety symptoms was observed in the add-on therapy group compared to the Sertraline + placebo after one month of administration (P = 0.039) (Table 4).‎

***Adverse Reactions***

Based on data from Table 5, no major and significant adverse effect or drug interaction was observed after Sertraline + Pasipy co-administration compared to the other group. The most remarkable side effect in Sertraline + placebo group was somnolence (F = 7, percent = 20.6%), which occurred more frequently in add-on therapy (F = 8, percent = 23.5%).

## Discussion

‘Fear appeal’ is a brain message against threatening situations (27). It is a distinguishing ‎characteristic in anxiety disorders (28) which persuades the suffered patient to do a warily action. ‎This could explain reduced omission errors after add-on therapy. Therefore, passion flower seems ‎to increase the positive risky behavior and remove hesitance features as expected. It is ‎accompanied by the Hamilton test results that reconfirm the potential effects of this herbal ‎medicine for GAD. Slight and non-significant prolongation in mean response time (RT) is ‎explained by relieving pathological impulsiveness, which is one of the most distinguished features ‎of GAD (29). Numerous studies revealed that GAD rarely achieves high end-state functioning at ‎post-treatment, and the influence of these treatments on quality of life is not quite proved (30). ‎Pharmacotherapy has been claimed the main stage of treatment. Despite advantages, one of the ‎concerns about the first-line medication is cognitive side effects (31, 32). Among ‎Benzodiazepines, which are known as one of the most promising medications, the difficulty in ‎discontinuing these medications is a crucial dilemma (1). CBT has been believed to be the most ‎effective treatment in GAD among the non-pharmacological management. Studies that consider ‎CBT have some limitations; for instance, the inter-personal differences and long duration of such ‎experiments can restrict reaching confirmed conclusions (33). The pharmaceutical industry relies ‎on plant-based medicines significantly

(34). ‎Passion flower and its active ingredients, chrysin and pyrone derivative maltol, are responsible for ‎the related CNS effects (35). Although the exact pharmacological mechanism is not fully known, ‎the majority of studies indicated that the sedative-hypnotic effects of passion flower are ‎presented through gama aminobutyric acid (GABA) neurotransmission (36). In a study by Appel ‎et al., passion flower was shown to antagonize GABAB receptor. However, ethanol site and ‎benzodiazepine site of GABAA receptors were not affected (37). Passion flower has been ‎demonstrated to be an efficacious drug for GAD management when compared with Oxazepam ‎and its undesirable side effects. The most preferences for anxiolytic effect of this phytotherapy ‎compared to the chemical medications are the venial impairment of performance (24), lack of ‎psychomotor dysfunction (38) or high sedation (39), which are promising in comparison with ‎psychiatric drugs with many of cognitive side effects (18, 19). The effects of cognitive function ‎have been reported in the literature. For example, in a study by Dimpfel et al., mathematical ‎calculation, concentration and memory tests were performed to evaluate the effects of passion ‎flower dry extract in a group of volunteers. The results showed no cognitive impairment even ‎though the psychometric scales were different from the RT test used in our study (40). Passion ‎flower 500 mg was administered before surgery and numerical rating scale (NRS) was utilized to ‎assess anxiety and sedation; besides, Trieger Dot Test and the Digit-Symbol Substitution were ‎used to evaluate psychomotor changes. The outcomes showed no significant difference in the ‎psychomotor function between the two groups after anesthesia (41). This study concluded that ‎passion flower does not affect reaction time, and therefore can be given to those patients whose ‎level of consciousness and speed of performance is important in their professional activities. In ‎our last trial, we found no adverse effect of passion flower on alertness in the healthy volunteers (25). However, small sample size and time limitation restricted our experiment. ‎

## Limitations

The limitations of the present study were as following: Firstly, the sample size was relatively small. Secondly, one month may not be considered long enough to precisely evaluate the effects of Passion flower extract. Thirdly, "structured interview", a more precise mean of evaluation of the patients, was not utilized in this study.

## Conclusion

This study noted that passion flower might be consumed as a safe (low side effects) add-on in the treatment of generalized ‎anxiety disorder. Further studies with longer duration are recommended to ‎confirm the results of this study.
